# System‐based strategies for p53 recovery

**DOI:** 10.1049/iet-syb.2017.0025

**Published:** 2018-01-15

**Authors:** Muhammad Rizwan Azam, Sahar Fazal, Mukhtar Ullah, Aamer I. Bhatti

**Affiliations:** ^1^ CASPR, Department of Electronics Engineering Capital University of Science and Technology Islamabad Pakistan; ^2^ Department of Bioinformatics and Biosciences Capital University of Science and Technology Islamabad Pakistan; ^3^ Department of Electrical Engineering National University of Computer and Emerging Sciences Islamabad Pakistan

**Keywords:** proteins, tumours, cancer, cellular biophysics, molecular biophysics, molecular configurations, biochemistry, differential equations, closed loop systems, bifurcation, biology computing, system‐based strategies, p53 recovery, systems theory‐based novel drug design approach, dynamic system, ordinary differential equations‐based mathematical model, control engineering practices, subsequent controller design, wild‐type p53, p53 revival, oscillatory, control system paradigm, mathematical model, Nutlin effect, attractor point analysis, domain‐of‐attraction, two‐loop negative feedback control strategy, cellular concentration, pharmacokinetic effects, cancerous cells, bifurcation analysis, p53 oscillation, anomalous cell

## Abstract

The authors have proposed a systems theory‐based novel drug design approach for the p53 pathway. The pathway is taken as a dynamic system represented by ordinary differential equations‐based mathematical model. Using control engineering practices, the system analysis and subsequent controller design is performed for the re‐activation of wild‐type p53. p53 revival is discussed for both modes of operation, i.e. the sustained and oscillatory. To define the problem in control system paradigm, modification in the existing mathematical model is performed to incorporate the effect of Nutlin. Attractor point analysis is carried out to select the suitable domain of attraction. A two‐loop negative feedback control strategy is devised to drag the system trajectories to the attractor point and to regulate cellular concentration of Nutlin, respectively. An integrated framework is constituted to incorporate the pharmacokinetic effects of Nutlin in the cancerous cells. Bifurcation analysis is also performed on the p53 model to see the conditions for p53 oscillation.

## 1 Introduction

The tumour suppressor protein, p53, is becoming a mainstream target of cancer research community due to its role in cancer suppression. In response to stresses or DNA damage, p53 induces multiple responses such as DNA repair, cell cycle regulation, senescence, and apoptosis [1]. Over the time, p53 signalling pathway has proven itself as one of the most promising and efficient mechanisms for combating this disease [2]. If a cell is endangered due to UV light, cellular stress, DNA damage, or hypoxia, p53 activates numerous pathways and initiates several responses in order to maintain the normal function of the cell [3]. Owing to this reason, p53 is sometimes rightfully called the guardian of genome [4].

MDM2, which is regulated by p53, degrades the level of p53 by its destruction [5], its production is also stimulated by p53, forming a feedback loop [6]. The MDM2 protein serves as an E3 ligase which degrades p53 by ubiquitination process. Although several other E3 and E4 ligases of p53 also exist [7, 8], but the E3 ligase MDM2 is the primary negative regulator of p53 [7, 8, 9, 10, 11]. MDM2 ubiquitination of p53 is either mono‐ or poly‐ubiquitination that negatively regulates transcriptional activity of p53. Nuclear export is triggered by mono‐ubiquitin, while nuclear p53 for degradation by the proteasome is triggered by poly‐ubiquitination. MDM2 marks p53 (by attaching a phosphate ion) for degradation by proteosome [12]. Overexpression of MDM2 regulates p53 through the negative loop and p53 cannot sustain its required level. Therefore, elevated level of MDM2 means deficiency of p53. When p53 protein cannot maintain its level, DNA damage and hence tumour growth cannot be taken care of. It makes MDM2 a streamline therapeutic target for cancerous cells retaining wild‐type p53 [13, 14].

p53 attaches to MDM2 at its designated binding site. The structure of p53 reveals that some small organic molecules can mimic the binding of p53 with MDM2, which could prevent the association of p53 with MDM2, leading to the elevated level of p53. Numerous small molecules are reported as repressors for the core regulation network of p53‐MDM2. Nutlin‐3a is one of such small molecule inhibitors which is already subjected to successful clinical trials. It binds to N‐terminal pocket of MDM2, preventing its bonding with p53 [13].

To investigate construction and deconstruction mechanism of p53 and efficacy of above‐mentioned approach, scientists have developed all kinds of mathematical models, including continuous time differential equations, discrete time differential equations, delayed differential equations, and stochastic models [15]. The effect of DNA damage on p53 pulses has been modelled by multiple researchers. Various mathematical models use different scenarios to create this pulsating behaviour. An ordinary differential equation (ODE)‐based model was built by Lev Bar‐Or *et al*. [16], this model considers a negative feedback between p53 and MDM2 which is responsible for oscillations. The model by Ciliberto *et al*. [17] displayed digital pulses in response to irradiation levels. The parameter set chosen in this model express limit cycles. The time, response stays in limit cycles is controlled by extent of DNA damage. Tyson *et al*. [18] used time delay model with negative feedback to generate damped oscillations.

Lipniacki *et al*. [19] proposed a hybrid model, which quantitatively analysed the experimental data. The DNA repair mechanism is built by considering variable duration in limit cycles with the help of Hopf bifurcation. Later on, they extended their work in [20] to incorporate pharmacokinetics of drug Nutlin. It is desired to choose a control‐oriented mathematical model which is controllable for the provided input. The model proposed by Hunziker *et al*. [21] offers a simplistic approach that allows control‐oriented analysis and drug dosage design, yet it includes all the major characteristics of p53 pathway.

## 2 p53 pathway mathematical model

**Fig. 1:**
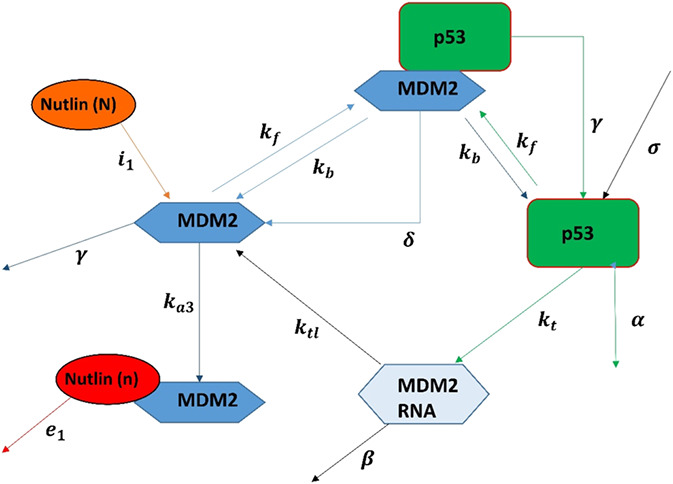
Integrated model of Nutlin PBK dynamics and p53 pathway dynamics

The model presented by Hunziker *et al*. [21] investigated the positive feedback loop of p53–MDM2 mRNA and negative feedback loop between p53 and MDM2, to produce oscillations in response. The schematic representation of the model is demonstrated in Fig. 1. The ODE‐based mathematical model is presented in (1)–(4)

(1) $$\frac{dp}{dt}=\sigma -\alpha p-k_{f}pm+k_{b}c+\gamma c$$

(2) $$\frac{dm_{m}}{dt}=k_{t}p^{2}-\beta m_{m}$$

(3) $$\frac{dm}{dt}=k_{tl}m_{m}-k_{f}pm+k_{b}c+\delta c-\gamma m$$

(4) $$\frac{dc}{dt}=k_{f}pm-k_{b}c-\delta c-\gamma c$$

The model mainly focuses on four concentrations, i.e. nuclear‐p53, *p*; Mdm2, *m*; Mdm2 mRNA, *m_m_*; and the p53‐Mdm2 complex, *c*. Table 1 lists the parameters of the model and their definitions.

**Table 1:** Definition of model rate constants and parameters [21]

Parameter	Definition	Value
σ	production rate of p53	1000 nM h^−1^
α	Mdm2‐independent deactivation/degradation of p53	0.1 h^−1^
δ	Mdm2 dependent deactivation/degradation of p53	11 h^−1^
*k*_t_	transcription of Mdm2	0.03 nM^−1^ h^−1^
*k*_tl_	translation of Mdm2	1.4 h^−1^
β	degradation rate of Mdm2 mRNA	0.6 h^−1^
γ	Mdm2 degradation/deactivation	0.2 h^−1^
*k_b_*	dissociation of Mdm2‐p53	7200 h^−1^
*k*_D_ = *k_b_*/*k*f	dissociation constant of Mdm2‐p53	1.44 nM

Dosage concentration and dynamics are defined by Puszynski *et al*. [20] with the help of physiological‐based kinetic (PBK) model. Puszynski *et al.* explored the pharmacokinetics data in mice to investigate the effect of Nutlin oral delivery. The following equation incorporates pharmacokinetic effects for Nutlin

(5) $$\frac{dN_{\text{tot}}}{dt}=p_{\text{oral}}D\delta_{1}e^{-\delta_{1}(t-t_{0})}-\delta_{2}N(N_{\text{tot}}),\;N_{\text{tot}}(t_{0})=0$$

where $N_{\text{tot}}$ is the total Nutlin comprising the Nutlin in plasma and in nucleus, $p_{\text{oral}}$ describes conversion from mg/kg to moles per distribution volume, *D* is the drug dosage (in mg/kg), and $t_{0}$ the initial time for drug delivery [20].

The effect of Nutlin dose on p53 pathway is simulated by integrating Hunziker *et al.* and Puszynski *et al.* models. The p53 dynamics are taken from Hunziker *et al.*, and Nutlin PBK dynamics from Puszynski *et al.*, reintegrated into Mdm2 rate (3). Nutlin is added as a sink term $k_{a_{3}}N$ as represented in the following equation

(6) $$\frac{dm}{dt}=k_{t}lm_{m}-k_{f}pm+k_{b}+\delta pm-\gamma m-k_{a_{3}}N(t)m$$

## 3 p53 pathway dosage design

Information contained in the cellular structure is insufficient to characterise complete behaviour of p53 pathway. Dynamics of the pathway are to be incorporated as well. Variation in parameters of MDM2–p53 loop can elicit multiple dynamic patterns such as damped oscillations, sustained oscillations, impulses, digital pulses, and bio‐modality [22].

Broadly, stresses invoke p53 in two types of responses, i.e. either oscillatory or sustained. For less extensive DNA damage, p53 pathway is reported to go into oscillations. The oscillations in the p53 pathway initiate further downstream targets that repair the cell by DNA repair, cell cycle arrest, or senescence [20, 23]. These oscillations consist of fixed pulse width and amplitude while their frequency is dependent upon the extent of damage. After each pulse of ∼6 h, DNA status is verified. In the case of DNA repair, p53 oscillations die out and the blocked cell cycle process is resumed.

**Fig. 2:**
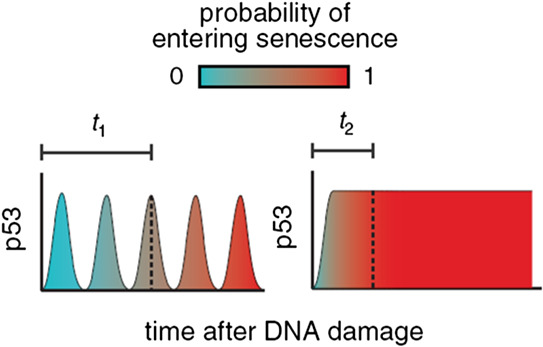
*Probability of entering senescence for pulsed and sustained p53 [* 24*]*

In response to extensive DNA damage, p53 demonstrates a sustained response, whose amplitude and width are dependent upon extent of DNA damage. Sustained p53 response expresses genes that induce senescence and leads to irreversible cell fate [24, 25]. Fig. 2 depicts the probability of entering senescence for pulsating as well as sustained p53. It can be seen that pulsed p53 provides extra time for DNA recovery, on the other hand sustained p53 accelerates senescence.

The effect of Nutlin on p53 pathway can be simulated in two ways for the determination of its dosage. The first dosage strategy is devised for a constant p53 response, and the other one is meant for inducing p53 oscillations.

### 3.1 Generation of constant p53 response

**Fig. 3:**
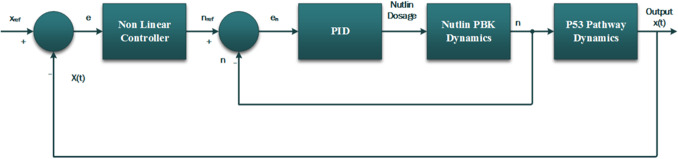
Block diagram of negative feedback control for Nutlin PBK dosage

For the production of a sustained p53 response, a two loop negative feedback strategy shown in Fig. 3 is employed. The outer loop comprises the p53–Mdm2 pathway and its non‐linear controller. This non‐linear controller determines the amount of Nutlin required in the pathway to revive p53. This required amount of Nutlin is termed as reference dosage or $n_{\text{ref}}$. Since our goal is to reduce Mdm2 as much as possible, so as to give some space to p53 for growth. Physiologically, it implies that the reference dosage of Nutlin required is determined by the non‐linear controller with the use of actual concentrations or states of the system. This gives the reference Nutlin dosage which should be present in the cell.

It can be rightfully assumed that for a cancerous cell, p53 level should be high and Mdm2 level should be reasonably low. The simplified version of p53 as given by (1)–(4) can be employed to determine the equilibrium point or an attractor point in the four‐dimensional state space consisting of the concentrations of p53, Messenger RNA, Mdm2, and p53–Mdm2 complex. To determine an attractor, (1)–(4) are solved with their left‐hand sides made zero. Since it will be a system of four non‐linear equations, there will be more than one solution. We will go for an attractor or equilibrium point that will have high p53 concentration and low Mdm2 concentration. Another condition is that the attractor has to be stable. The stability can be determined by looking at the eigenvalues of the Jacobian of (1)–(4) with respect to states evaluated at the equilibrium point. Using these two conditions, the following attractor is found suitable:

$$x_{0}=\begin{array}{cccc} {}[64.0169 & 211.3598 & 4.9701 & {90.318]}^{T} \end{array}$$

where $u_{0}=197.1297$. Once this equilibrium point is determined under the above conditions, then it would be desirable to drive the system (1)–(4) to this equilibrium point in order to revive p53. In order to do that, we need to determine how far is the system from this equilibrium point in the four‐dimensional state space. From the system consisting of (1)–(4), the system states at any given time *t* can be defined as:

$$x(t)=\begin{array}{cccc} {}[p & m_{m} & m & {c]}^{T} \end{array}$$

The output of the closed‐loop system is complete state vector x(t) which is fed back to the non‐linear controller. The difference between actual state vector ***x*** (*t*) and the desired state vector $x_{\text{des}}$ is the error to be minimised. Here, $x_{\text{des}}$ is equal to the attractor point $x_{0}$. The control objective is to derive the system trajectories to this desired equilibrium point from arbitrary initial trajectories

(7) $$e=x_{\text{des}}-x(t)$$

The half of square of the Euclidean norm can be taken as Lyapunov candidate function:

(8) $$E=\frac{1}{2}e\dot{e}$$

This *E* belongs to $R^{4}$, taken as a measure of the cell on how far it is from p53 revival. Ideally, we would like this measure to be driven to zero so that the p53 in the cell gets active. This driving of the cell will be achieved by the recommended dosage of Nutlin in the cell termed as $n_{\text{ref}}$. The mechanism for the computation of this variable or the controller design is elaborated next. Using Lyapunov theory, it is known that the system (1)–(4) will reach the desired equilibrium, if the derivative of *E* is negative. The derivative of *E* can be figured as:

(9) $$\dot{E}=e^{T}\dot{e}$$

which comes out to be:

(10) $$\dot{E}=[e_{1}\dot{p}+e_{2}{\dot{m}}_{m}+e_{3}\dot{m}+e_{4}\dot{c}]$$

Substituting the values from (1) to (4) in the above equation and making $\dot{E}=0$, the reference dosage or the control turns out to be:

(11) $$n_{\text{ref}}=\left(\frac{1}{e_{3}k_{a_{3}}m}\right)(e_{1}\dot{p}+e_{2}\dot{m}m+e_{3}\bar{m}+e_{4}\dot{c})$$

where

(12) $$\bar{m}=k_{tl}m_{m}-k_{f}pm+k_{b}c+\delta c-\gamma m$$

It is assumed that the system is away from its desired state so that the denominator does not become zero. In order to make *E* negative definite, we add a term kϵ in $n_{\text{ref}}$:

(13) $$n_{\text{ref}}=\left(\frac{1}{e_{3}k_{a_{3}}m+k\epsilon }\right)(e_{1}\dot{p}+e_{2}\dot{m}m+e_{3}\bar{m}+e_{4}\dot{c})$$

This term will drive the system to the desired equilibrium point asymptotically. Where ϵ is a very small number, i.e. |ϵ|→0 used in the simulations to avoid singularity, which can come due to the error $e_{3}$ in the denominator of (13). The coefficient *k* can be tuned during the *in silico* trials. If we had any performance issues, then we could have used exponential stability argument to determine $n_{\text{ref}}$.

Owing to the presence of $e_{3}$ in the expression for $n_{\text{ref}}$, the system can be taken arbitrarily close to the desired point, i.e. in a ball of small radius around the equilibrium point. Once the system is in the ball then the control is made equal to $u_{0}$ to make it go to the equilibrium point.

To maintain $n_{\text{ref}}$ in the cell, a negative feedback loop is devised for the PBK dynamics of Nutlin [26]. The dosage given to the patient should be a function of the error which comprises the difference between the desired Nutlin concentration and the actual Nutlin concentration present in the cell, as determined by the above stated PBK dynamics. The error is defined as:

(14) $$e_{n}=n_{\text{ref}}-n$$

where *n* is the actual amount of Nutlin present in the cell. Both the loops are shown in the accompanying Fig. 3. On this error, a proportional, integral, and derivative (PID) controller is tuned, so that the error goes to a very small value in minimum time. The dosage *D* is given as:

(15) $$D=K_{p}+K_{i}\int e_{n}\;dt+K_{d}\frac{d}{dt}e_{n}$$

where $K_{p}$, $K_{i}$, and $K_{d}$ are the proportional, integral, and derivative gains of PID controller. The proportional–integral (PI) control is used to minimise the error between $n_{\text{ref}}$ and *n*, objective is to achieve zero steady‐state error. The derivative control term is used to get a smooth response (by minimising the oscillations) and to speed up response of the inner loop. In the cascaded control arrangement, both the loops are running simultaneously, where the reference dosage is being generated by the outer loop and inner loop tracks this reference dosage keeping in view the cellular dynamics of the drug. The inner loop should be fast as compared to the outer loop, so that the reference value of the inner loop can be considered relatively constant.

#### 3.1.1 Results and discussion

**Fig. 4:**
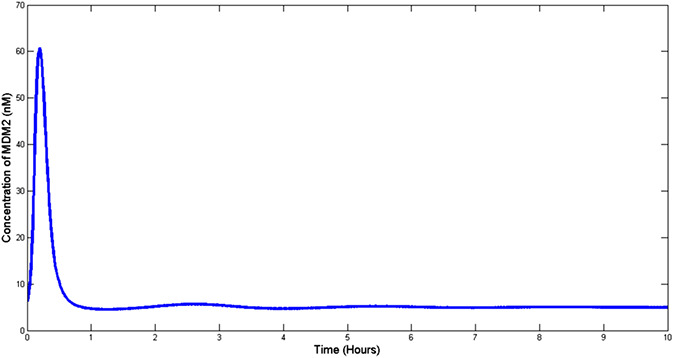
Mdm2 reduced to a minimal level by the action of Nutlin

**Fig. 5:**
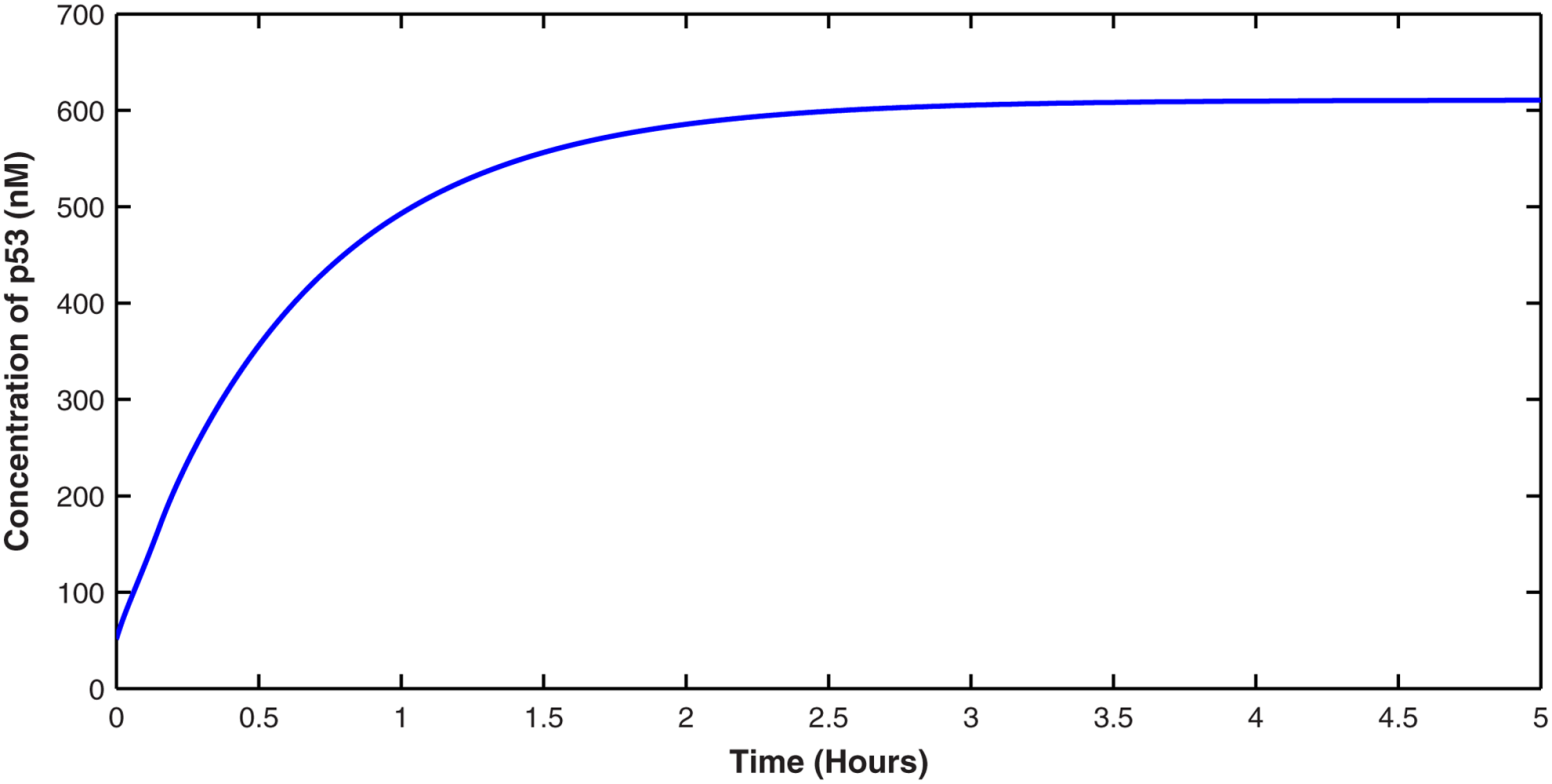
Sustained level of p53

**Fig. 6:**
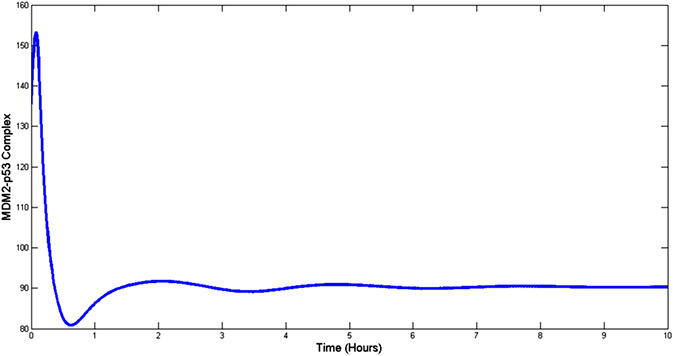
p53–MDM2 complex concentration

**Fig. 7:**
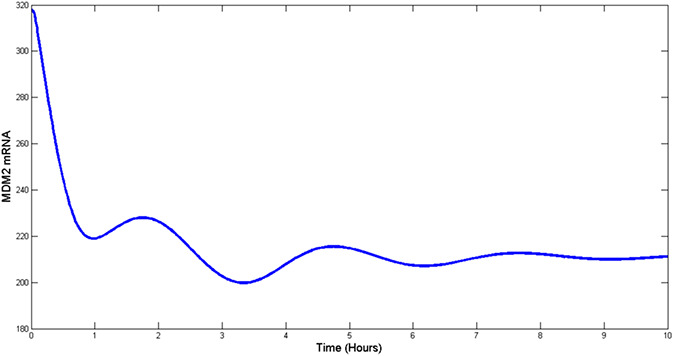
MDM2 mRNA concentration

**Fig. 8:**
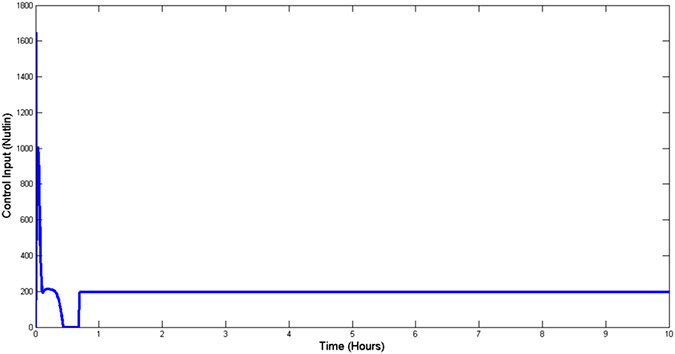
Control input (Nutlin)

The effectiveness of the proposed control scheme is evaluated by closed‐loop simulations tests shown in Figs. 4, 5, 6, 7. It is evident that the concentrations of all state variables are reaching their desired equilibrium values. The convergence time is within an hour, this may be too quick as suggested by the high overshoot in Nutlin dosage represented in Fig. 8. However, this may be reduced by tuning the gains of controllers as per therapeutic requirement. After passing initial bump, Nutlin remains near 200 mg/kg which is in accordance with the experimental results conducted by the authors in [27, 28, 29]. They used intravenous and oral drug delivery in the range of 10–400 mg/kg in various range of intervals. A downward bump in Nutlin dosage can be seen at around 1 h. This is because of the fact that at this point, $e_{3}$ becomes small and to stop the controller effort from diverging the controller action is set to zero. Later on, it takes on the equilibrium input value.

From the drug‐manipulation viewpoint, it is evident that the drug is being constituted by the contribution of each state variable. The controller action is based on different mathematical operations being performed on the variables, namely addition, subtraction, scalar multiplication, integration, and differentiation. The physical realisation of these operations becomes important when implementing the proposed closed‐loop control system for drug delivery in the human body.

**Fig. 9:**
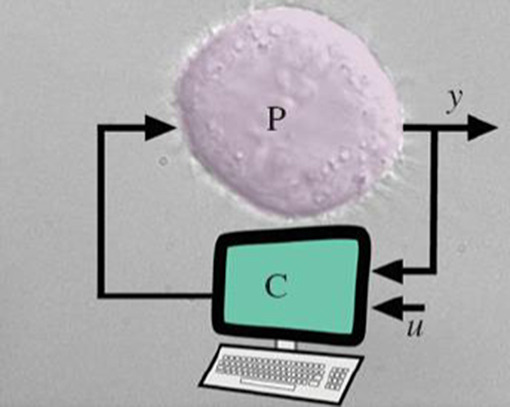
In silico feedback control implementation

**Fig. 10:**
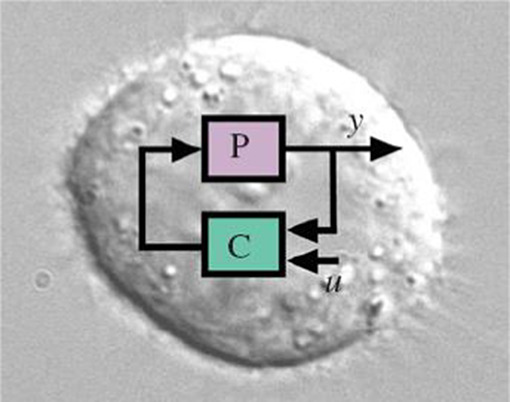
In vivo feedback control implementation

Realisation of a feedback control system demands high‐quality sensing, adequate computational power, and accurate actuating components. This becomes more challenging when we are dealing with biological systems. Engineers and experimental biologists have tackled this challenge in two ways: (i) *in silico* feedback control and (ii) *in vivo* feedback control, illustrated in Figs. 9 and 10, respectively.

*In silico* control implementation considers the molecular circuitry of a cell or population of cells as the process ‘P’ to be controlled, and the controller ‘C’ is implemented on a computer, outside the body, as depicted in Fig. 9. For the real‐time implementation, techniques like microscopy [30, 31], flow cytometry [32], and rapid immunoassay [33] enable fast measurements of protein concentration in patients. Methods like immunomagnetic‐ electrochemiluminescent require seconds to collect samples from patient's serum and minutes to complete the measurement process. The sampling period between intervals can be set according to the therapeutic requirements.

The measured data ‘*y*’ is compared with the desired data ‘*u*’ *in silico* and the control input computed by the controller serves as the dose for targeted cells. The interface between computer and cells is achieved by biological transducers that are capable of responding to input in either light or chemical form. The proposed control scheme for p53 protein revival can be implemented in this manner, provided all the state measurements are available. The implementation becomes more challenging for the inner loop controller as it requires at every instant the measurement for the drug Nutlin inside the cell. A better way to tackle this problem is to implement the controller inside the cellular structure.

Synthetic biology enable engineers to programme living cells to serve as therapeutic agents to cure genetic disease. Engineered bio‐systems are built with a bottom‐up approach of synthesising small parts which constitute functional modules, and composition of these modules build systems [34, 35]. *In vivo* feedback control employs synthetic biology to control the cellular behaviour by assembling molecular circuits in cells [36]. Both the process and controller are realised within cells with the help of bimolecular processes, as depicted in Fig. 10.

**Fig. 11:**
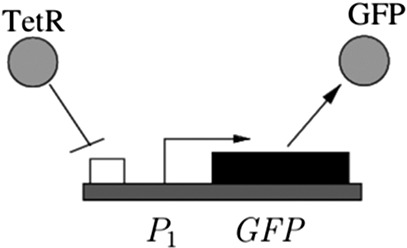
Genetic implementation

**Fig. 12:**
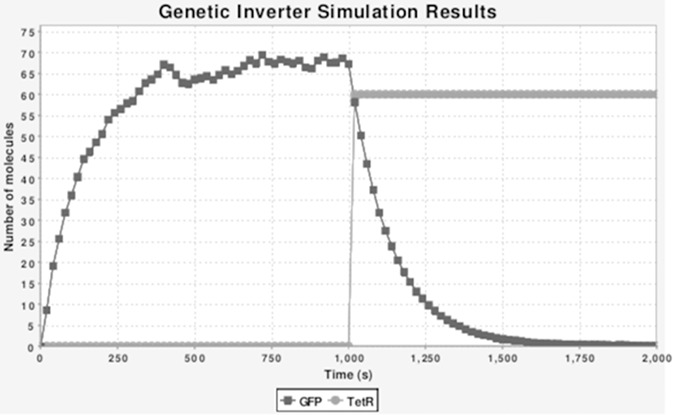
Stochastic simulation

With the help of genetic circuits and synthetic sensors, any feedback control system can be implemented in to cells. The transcription of a gene is initiated and regulated by transcription factors (TF). The binding cites of TF can be used in designing synthetic systems [37]. For example, a basic logic gate ‘inverter’ can be constructed from genetic material as shown in Figs. 11 and 12. Genetic circuit is composed of a promoter and gene which transcribes green fluorescent protein (GFP). Cells containing this circuit glow green whenever input protein TetR is unavailable in the cell [38]. There are number of regulators that control the rate of gene transcription by binding to separate gene promoter regions. Many logic gates have also been constructed with these DNA‐binding proteins [39].

Genetic implementation of negative feedback have been achieved by either increased degradation of mRNA [40] or suppression of translation process by making use of mRNA binding proteins [41]. Chemical reactions are employed to construct and implement integral feedback control [42] and non‐linear quasi‐sliding mode control [43]. However, all of these techniques require rigorous theoretical analysis to ensure stability, reliability, and robustness. We therefore propose a hybrid method of controller implementation. The non‐linear outer loop Lyapunov controller will be embedded inside a digital computer (*in silico*) and the inner loop will be synthesised by biological circuits (*in vivo*).

### 3.2 Inducing p53 oscillations

The negative feedback loop between MDM2 and p53 unfolds oscillatory dynamic behaviour [18], specially whenever less significant DNA damage occurs. According to [44, 45, 46] in response to stresses, p53 repairs the cells by exhibiting sustained oscillations. Moreover, when DNA damage stress is inflicted, it causes γ to increase and δ to decrease [21].

Research work carried out by Eliaš *et al.* in [47, 48] discuss the possibility of limit cycles, which can be the baseline cause for the p53 oscillatory behaviour. Inducing p53 oscillations of a particular frequency and pulse width requires modification in the system parameters which cannot be achieved by feedback control. Instead, the effect of Nutlin may be simulated by varying the values of parameters Mdm2 deactivation constant γ and Mdm2‐dependent p53 deactivation constant δ. It is worth noticing that according to Hunziker *et al*. [21], γ is the most sensitive parameter in the whole model as well. The effect of parameter variation is observed by performing simulations for three different cases, 1000 h each, for which decay does not occur unless otherwise stated.

#### 3.2.1 Variation in γ only

γ is varied in between 0.2 and 0.38 (further increase in γ causes simulations to be unstable), it is observed that p53 mean value varies from 17 to 26 nM and p53 peak‐to‐peak value increases from 2 to 45 nM. The oscillations have pulse width of around 4 h.

#### 3.2.2 Variation in δ only

When δ is decreased from 11 to 4, it is observed that p53 mean value increases from 17 to 30 nM and p53 peak‐to‐peak value increases from 2 to 30 nM. The oscillations have pulse width of around 4 h except for the case of δ=8 where oscillation decay is observed.

#### 3.2.3 Decreasing δ and increasing γ simultaneously

A decrease in δ and an increase in γ exhibits an increase in the mean p53 value and peak‐to‐peak p53 value. Pulse width remains the same as for previous cases.

Clearly, the decrease in δ and increase in γ are responsible for increased oscillations. It is worth noticing that the oscillation period is constant (≃4 h) for any case, suggesting a possibility of limit cycle. Puszynski *et al*. [20] suggest p53 oscillations of ≃6 h time period, indicating a limit cycle.

Cells show undamped oscillations of around 6 h in response to DNA damage, these long trains of undamped oscillations propose that p53 pathway is capable of producing limit cycle oscillations [49]. Indicating that the equilibrium point should be a Hopf bifurcation point, presumably, it will be a stable limit cycle. The Andronov–Hopf bifurcation theorem states that, for the existence of Hopf bifurcation, there should exist one pair of complex eigenvalues (all other eigenvalues being real and negative) which crosses the imaginary axis as the bifurcation parameter γ is changed. In the oscillating mode, equilibrium points are moved to

$${\left[\begin{array}{cccc} p & m_{m} & m & c \end{array}\right]}^{T}={\left[\begin{array}{cccc} 19.648 & 19.303 & 6.475 & 90.73 \end{array}\right]}^{T}$$

When γ is increased from its nominal value of 0.2 to γ=0.25815, the eigenvalues were found to be:

$$\left[\begin{array}{c} \begin{array}{cccc} -141556.81 & -3.5222 & 0.000002+i1.88 & 0.000002-i1.88 \end{array} \end{array}\right]$$

**Fig. 13:**
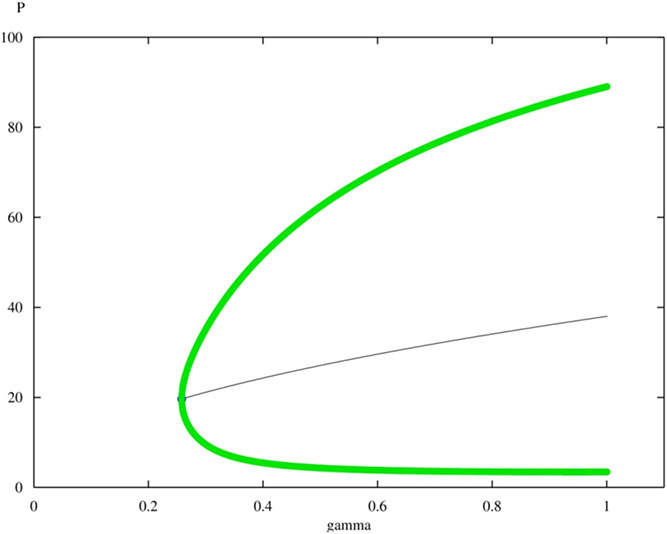
*Hopf bifurcation in p53 by changing gamma (* γ *)*

In our previous work in [50], Hopf bifurcation diagrams were computed in p53 by varying γ and found that increasing γ from its nominal value of γ=0.2 to γ=0.25815 drives the overall response towards Hopf bifurcation point represented in Fig. 13.

## 4 Conclusion

The work demonstrates a system‐oriented framework for devising dosage design strategy for p53 pathway. A control‐oriented mathematical model is considered with an addition of PBK dynamics for small molecule drug Nutlin. The integrated model is used to achieve a drug dosage strategy for reactivation of wild‐type p53. The effect of Nutlin can be simulated in two ways. The first dosage strategy is devised for a constant p53 response, and the other one is meant to induce p53 oscillations.

The problem is defined in the control system paradigm where two loop feedback control strategy is employed to produce sustained response of p53. The outer loop comprises the p53–Mdm2 pathway and its non‐linear controller. The non‐linear controller determines the required amount of Nutlin, i.e. reference dosage. However, to maintain reference dosage in the cell, a negative feedback inner loop is devised for the PBK dynamics of Nutlin. The PID control provides a dosage which is function of the error between reference and the Nutlin present in the cell. It is shown by *in silico* trials that sustained response of p53 can be achieved by proper drug administration. The obtained dosage remains within suitable limits.

However, mere application of feedback controller is not sufficient to obtain oscillatory response as dissociation of p53–Mdm2 complex is required. The oscillatory behaviour can be treated as limit cycle. With the help of Hopf bifurcation theory, Mdm2 degradation constant is found to have ability to drive p53 in to oscillatory mode.

Hence, it can be concluded that all p53 responses can be achieved by proper administration of Nutlin dosage. Feedback control being a generic approach can be applied to other similar pathways as well to obtain required therapeutic drug dosage.
